# Novel Wearable Device for Blood Leakage Detection during Hemodialysis Using an Array Sensing Patch

**DOI:** 10.3390/s16060849

**Published:** 2016-06-09

**Authors:** Yi-Chun Du, Bee-Yen Lim, Wei-Siang Ciou, Ming-Jui Wu

**Affiliations:** 1 Department of Electrical Engineering, Southern Taiwan University of Science and Technology, Tainan 71005, Taiwan; da320209@stust.edu.tw (B.-Y.L.); ma32d201@stust.edu.tw (W.-S.C.); wmr216@yahoo.com.tw (M.-J.W.); 2 Department of Internal Medicine, Kaohsiung Veterans General Hospital Tainan Branch, Tainan 71051, Taiwan

**Keywords:** hemodialysis (HD), array sensing patch, wearable device, risk level monitoring

## Abstract

Hemodialysis (HD) is a clinical treatment that requires the puncturing of the body surface. However, needle dislodgement can cause a high risk of blood leakage and can be fatal to patients. Previous studies proposed several devices for blood leakage detection using optical or electrical techniques. Nonetheless, these methods used single-point detection and the design was not suitable for multi-bed monitoring. This study proposed a novel wearable device for blood leakage monitoring during HD using an array sensing patch. The array sensing patch combined with a mapping circuit and a wireless module could measure and transmit risk levels. The different risk levels could improve the working process of healthcare workers, and enhance their work efficiency and reduce inconvenience due to false alarms. Experimental results showed that each point of the sensing array could detect up to 0.1 mL of blood leakage and the array sensing patch supports a risk level monitoring system up to 8 h to alert healthcare personnel of pertinent danger to the patients.

## 1. Introduction

The 2015 United States Renal Data System (USRDS) annual data report showed that chronic kidney disease (CKD) is continuously receiving more attention. This led to the promotion of the National Kidney Disease Education Program (NKDEP) by the National Institute of Diabetes and Digestive and Kidney Diseases (NIDDK) beginning as early as 2002 in order to provide information for patients and care providers regarding the detection of CKD, as well as information on how to take care of people with the disease. This program has obtained the support of the Centers for Disease Control (CDC) since 2007 and has reported on many aspects of this important chronic condition. Its latest report showed that Taiwan ranked first in the world (458 people per million) and has the highest incidence of treated end-stage renal disease (ESRD). It is followed by Mexico with 421 people per million and the US with 363 people per million. Subsequently, the report also showed that hemodialysis (HD) remains the world’s primary means of treating end-stage renal disease. There are two types of treatment, namely hemodialysis and peritoneal dialysis. In about 80% of the countries, more than 80% of end-stage renal disease patients choose HD as the primary treatment [1].

In HD treatment, venous needle dislodgement (VND) is a major concern in healthcare systems worldwide. A survey showed that approximately 44,000 to 98,000 Americans die annually due to adverse events in healthcare. For this safety issue, MacRae *et al.*’s study showed that during HD, incidence of VND from the fistula site has an annual growth rate of 5.2% [2]. It carries a serious implication and several literatures on medical risk management elucidate VND as a potentially serious complication in HD. If the blood pump is not stopped immediately, the patient could bleed to death within minutes [3]. As Ribitsch *et al*. [4] reported in their study, the American Nephrology Nurses’ Association (ANNA) carried out an investigation on VND and its consequences, and results showed that 77% of nurses had seen at least one incidence of VND in the past five years.

At present, there are commercially available products that could be used for VND blood leakage detection; one such products is New Zealand’s Anzacare HEMOdialert™ [5]. This product is basically based on the changes of the voltage signal in the sensor. It includes a detector with two spaced apart electrodes, and each electrode is connected to a signal-generating source via a lead. Since the device is made of plastic material, penetration of blood or other fluid would not damage it. It can be reused by cleaning with alcohol or other antibacterial disinfectant and needs to be replaced when the wire is bent (about six to nine months). The VND Alarm [6] made by Sweden’s Redsense Medical Company uses an array sensor for optical fiber transmission and reception. It uses the optical method as its detection method and has a measurement sensitivity of 1 mL. When VND blood leakage occurs, blood blocks light transmission, thereby activating the warning device. The monitoring sensor increases the safety level for HD patients, and reduces the potential risk in the clinics. Ahlmén *et al.* [7] reported in their study that the Redsense blood leakage detector was tested in 200 HD treatments, and nurses indicated that its use significantly improved the safety of the HD therapy. However, since optical fiber probes and needles must be parallel during usage, this could easily lead to needle compression and dislocation, causing a false alarm. Excessive compression may also influence dialysis treatment and even harm patients.

Wolff *et al*. [8] filed a patent on a VND indicator. The apparatus includes a line shut-off and a pressure sensor. The infusion pressure curve between the circulation device and the patient’s blood vessel during dialysis indicates the incidence of needle dislodgment. Lay-Ekuakille *et al.* [9] presented an alternative method by observing the changes in the dynamic pressure generated by the flow of intravenous fluid to detect whether the patient experiences VND blood leakage. In 2015, Chuang *et al.* proposed a bracelet monitoring device that includes a photo-interrupter, a Bluetooth 4.0 wireless module, a power supply, and alarm components. The absorbent material is placed at the light sensing position of the photo-interrupter, which causes the received light intensity to change during blood leakage. Once blood leakage occurs, the absorbent material absorbs the blood due to the capillary action and triggers the alarm signal. Then the signal is transmitted to a monitoring computer terminal via a Bluetooth wireless transmission. The photo-interrupter method can achieve a sensitivity of 0.1 mL. It is able to detect small amounts of blood leakage in order to avoid possible infection [10].

However, the above-mentioned products are costly for most patients, which hinders their popularization in the market. Subsequently, all products require 1 to 2 mL of blood to trigger blood leakage detection due to their sensor design [5,6,7,8,9]. Previous studies have elucidated the use of single-point sensing that requires a specific arrangement of the sensors, where a misalignment would make it prone to false alarm notifications. Thus, Sweden’s Redsense Medical Company also stressed the importance of a constant reminder to pay attention to the sensor placement [6]. Therefore, this study proposed a wearable device for blood leakage detection that uses a self-designed array sensor patch to provide a wide detection area and reduce the occurrence of false alarms. This method aims to detect a general VND blood leakage during HD and also to detect small blood leakage that may cause infection. For clinical use, the array sensor patch in this research will use a mapping circuit to evaluate different risk levels. When blood leakage occurs, blood will start leaking from the vicinity of the needle’s tip. When the sensing point farther from the tip of the needle detects a signal, it means that the patient’s blood has widely spread and is considered to be a high risk level. On the contrary, if the signal is detected from the sensing point closer to the tip of the needle, it is classified as a lower risk level. A low risk level could represent minor bleeding that can be dealt with easily. The different risk levels can improve the working process of healthcare workers, and enhance their work efficiency and reduce inconvenience due to false alarms. Nowadays, most of the products or research utilize wired transmission or Bluetooth wireless transmission for data transmission. However, Bluetooth has its limitations when applied to multi-bed monitoring. Subsequently, Wi-Fi is one popular technology for wireless network connections that involves relatively short-range communication between radios, where one radio operates in a client device and the other operates in a network infrastructure endpoint device such as an access point (AP) or router. Many computing devices include Wi-Fi radios as standard equipment. Nonetheless, the power consumption would be slightly higher compared to other wireless methods. It is then imperative to properly design the transmission process and reduce unnecessary power consumption. Thus, this study integrates Wi-Fi wireless transmission and access points (AP) structure to achieve multi-bed monitoring. When a high risk level is detected, it immediately activates the alert system on the wearable device composed of light and sound. At the same time, the warning signal is transmitted to the system database and synchronized to the monitoring terminal at the nursing station through Wi-Fi and a notification display alerts healthcare workers to immediately take appropriate action to prevent fatal incidents.

## 2. Materials and Methods

### 2.1. Architecture of the Blood Leakage Detector

**Figure 1:**
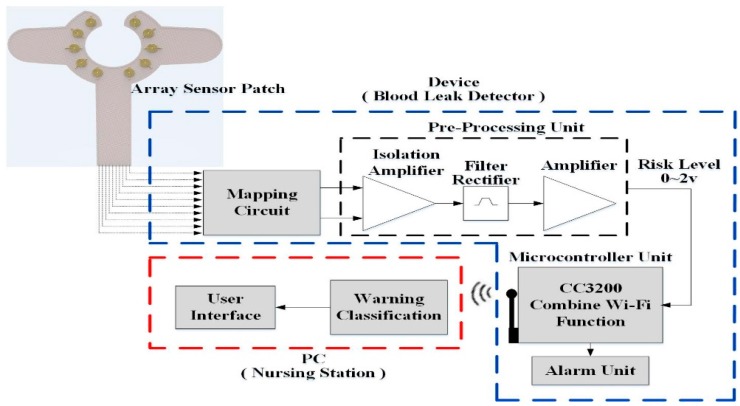
System architecture.

A novel design for a wearable blood leakage detector includes the array sensor, the mapping circuit, the pre-processing and microcontroller unit and the alarm unit. Figure 1 shows the system architecture diagram. The array sensor made by flexible printed circuit board (FPCB) had 10 sensor points on it to provide a wider detection area. The pattern design of the sensor array patch was discussed with clinicians and nurses and it was agreed that the main point of consideration is that the sensor should be able to surround the puncture point of the needle and that it should be easy to set up on the patient’s hand. The puncture point of the needle is also the leakage point location. Moreover, the clinicians hope that the sensor does not cover the puncture point directly to reduce the possible risks when replacing the sensor during hemodialysis, whenever necessary. The final pattern and shape are shown in Figure 1. All the sensor points were covered with gauze on top. The different weights could produce different signals that go through signal pre-processing to perform voltage stability, amplification and convert the multiple voltage inputs into a single voltage output with a range of 0–2 V to determine the risk level. Further, through an asynchronous Wi-Fi module, the internal micro control unit (MCU) determines the threshold analysis. When the risk level exceeds the threshold, the module triggers an alarm immediately. Finally, data are sent through Wi-Fi to the nursing station for evaluation. Detailed internal functions are as follows:

### 2.2. Blood Leakage Detector Components

#### 2.2.1. Array Sensor Patch and Mapping Circuit

**Figure 2:**
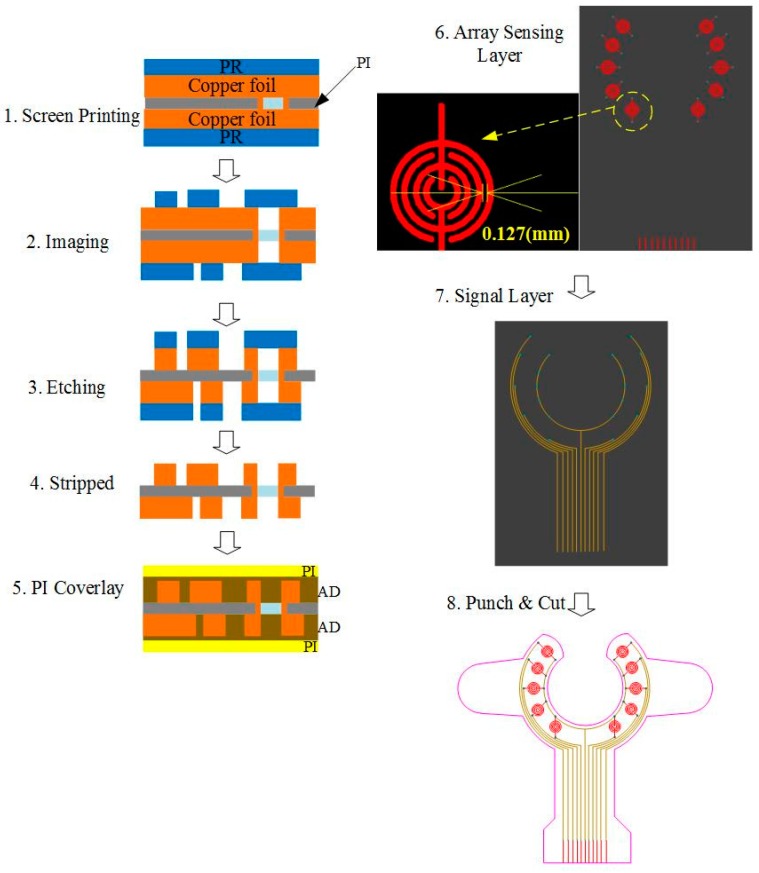
Flowchart for array sensor production.

The proposed device uses an array sensor combined with a mapping circuit and a Wi-Fi module to create a wearable VND blood leakage detection device during HD. The array sensor patch is made of FPCB, and the sensor development process is shown in Figure 2. First, the array sensing point and signal transmission line were screen-printed on the substrate. The signal line was embedded in the insulating layer during the production process with only the array sensing point protruding outside. The signal line is insulated in order to avoid signal transmission interference. Since the FPCB was produced by adopting imprinting technology, the interval surrounding of the sensing point of the line could reach up to 0.1 mm. Such an approach could prevent a false alarm during detection. In addition, a single sensing point possesses the best sensing sensitivity (<0.1 mL blood). The detailed design and circuit architecture can be obtained from [11]. Furthermore, imprinting technology makes it possible for changes in the sensing point or transmission line according to user demand at the same cost.

**Figure 3:**
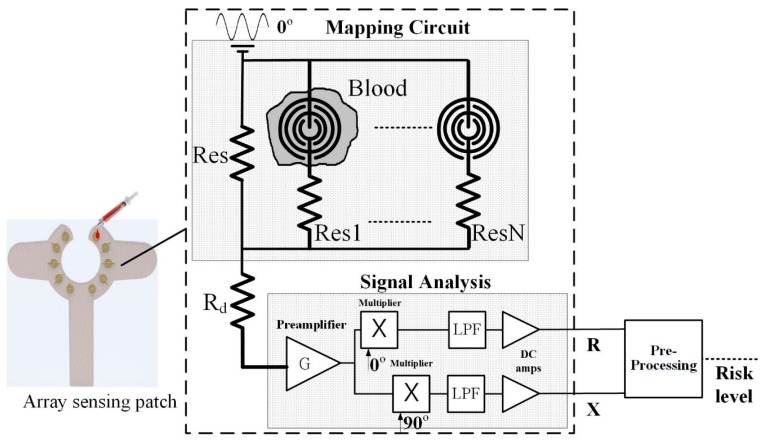
Mapping circuit and pre-processing schematic diagram [11].

After the sensing point detects blood leakage, the mapping circuit assigns the weight ratio of each conduction point and converts the multiple voltage inputs into a single voltage output that is called the risk level. After pre-processing the signal, signal analysis is performed in the MCU to achieve blood leakage classification. The design of the mapping circuit and pre-processing schematic diagram is shown in Figure 3. The risk level is obtained using Equation (1).

(1) $$\mathrm{Risk}\;\mathrm{level}=\sqrt{R^{2}+X^{2}}$$

#### 2.2.2. MCU and Wi-Fi Module

In order to improve Bluetooth limitations in multi-bed monitoring, this study used a combined MCU and Wi-Fi transmission module, CC3200 (Texas Instrument, CC3200 Simple Link include Microcontroller subsystem), for blood leakage detection and wireless transmission. This module includes a Wi-Fi network processor, a power management unit, and a microcontroller unit. This module has the advantages of low voltage requirements of only 2.1–3.7 V and small size (18 × 30 mm). Subsequently, this module uses 802.11 b/g/n protocols, which have an effective indoor range of up to 100 m according to different specifications of antenna configurations.

#### 2.2.3. Alert Components

Aside from using Wi-Fi wireless transmission to send messages to the nursing station, the built-in screen and sound capabilities of the device can be synchronized to provide visual and audible warning functions. The descriptions of the alert components are as follows.

- OLED Graphic Display: The device proposed in this study, a 0.96” 128 × 64 organic light-emitting diode (OLED) is used in the blood leakage detector to show the device’s power level and the visual warning signal. Compared to conventional LCD panels, OLED has the advantages of high brightness, flexibility, and wide viewing range which saves the overall circuit space of the detector. During dialysis, it could provide users with an immediate warning signal and shows the device’s power level [12,13].
- Buzzer: A buzzer as an integrated electronic signal warning device has been widely used in medical devices. It is classified into two types: electromagnetic and piezoelectric element. We used the smaller-sized electromagnetic buzzer as the sound warning device. When used clinically, it may produce noise and disturb other dialysis patients; therefore, this device is designed with sound alert on/off features and can be used according to user preference.

#### 2.2.4. Package and Wearable Device

**Figure 4:**
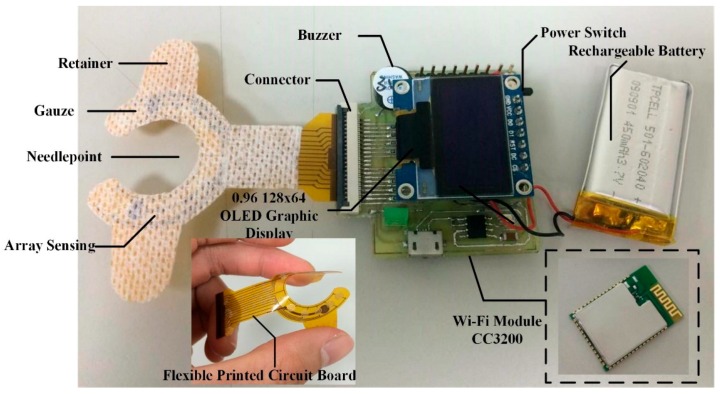
Photograph of the blood leakage detector.

The outer case of the device developed was produced using three-dimensional (3D) printing and polylactic acid material, with a size measurement of 35 mm × 35 mm × 15 mm as shown in Figure 4. The Wi-Fi module is utilized to replace the traditional USB data cable. Further, it could be adhered directly to the body surface of the patient without discomfort. When the dialysis patient experiences blood leakage, a real-time alert is produced and, at the same time, data will be transmitted wirelessly to the database system for assessment of the risk level that can be displayed on the nursing station monitoring terminal interface.

### 2.3. Signal Processing and User Interface

**Figure 5:**
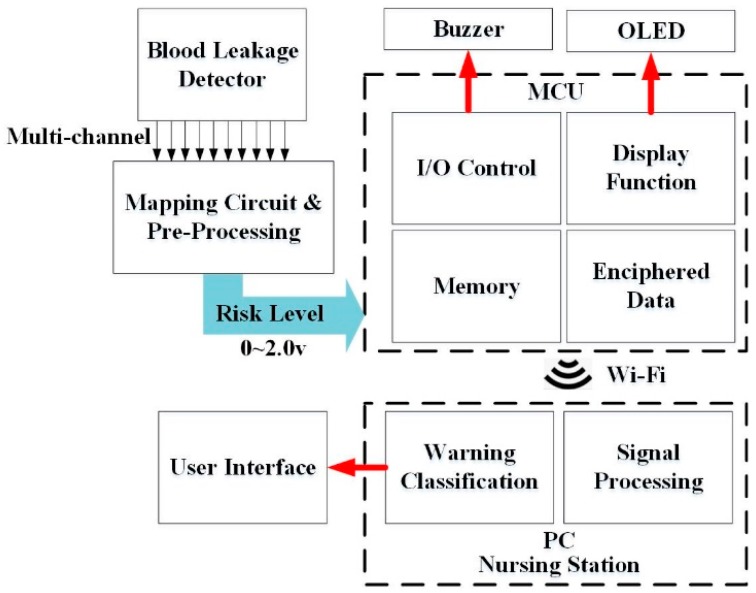
Signal processing flowchart.

Figure 5 shows the signal processing flowchart. After the array sensor detects a blood leakage, the signal goes through the mapping circuit and pre-processing to produce an output risk level signal with a value between 0–2 V. Subsequently the AD value enters the MCU to verify by threshold analysis and utilizes the OLED or buzzer to manifest both auditory and visual displays. The research adapts an asynchronous Wi-Fi transmission that adjusted three modes of operation depending on different risk levels. In the normal conditions, when the risk level is under 0.5, the wireless transmission is switched into the -saving mode. In the warning condition, when the risk level is between 0.5 and 1, it means that the patient has a small amount of blood leakage and the transmission function is turned on with the transmission frequency set to 1 Hz. In the critical condition, when the current risk level is higher than 1, the transmission frequency is set to 10 Hz and also will activate the audio and visual alarms of the device. Through this gradient operational method, it can effectively reduce the amount of data transferred and concurrently saves power.

### 2.4. System Design of the Circulation Simulation Test

**Figure 6:**
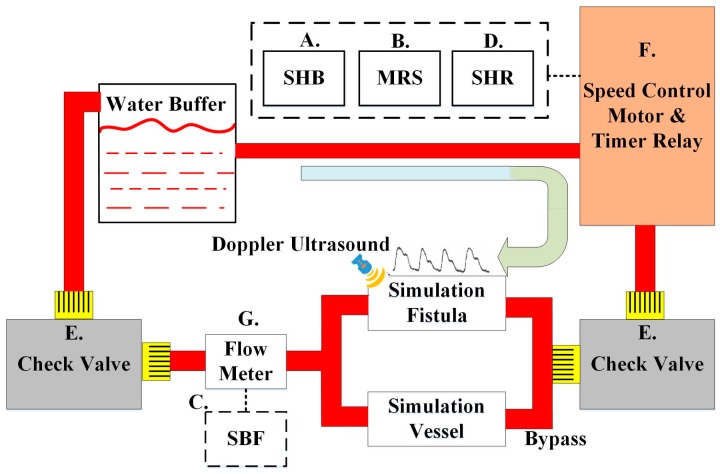
System block diagram for blood circulation simulation.

In order to verify the feasibility of the device in actual clinical use, this study designed a set of human blood circulation simulation systems. The goal is to test different blood flow rates during dialysis and detect the device’s sensitivity in detecting blood leakage. The system incorporates a speed control motor that could simulate the flow of blood in the fistula of the punctured arm model. This model mimics the arm of a real patient, which simulates the skin, fistula, veins and arteries (GENERAL DOCTOR, AA2429, Shanghai, China). A hand-held Doppler ultrasound (KOVEN, Bidop^®^ 3 Vascular Ultrasound Doppler, Winnipeg, MB, Canada) with Smart-V-Link™ software was used to test the consistency of the simulated fistula blood flow data with that of the actual human body. The extracorporeal circulation simulation of Saucedo-Zeni N *et al.* was used for the system design [14]. Figure 6 is the human blood circulation simulation system design block diagram. A five-digit display (TMA Technology Corporation, DMA-A, Kaohsiung, Taiwan) was used as the display device, where (A) is the simulation heartbeat (SHB) and displays the SHB generated by the system from the start to the end. A self-designed pulse-counting module was used for the sensing device, and similar waveform detection methods were used to accurately count each SHB and reduce noise; (B) shows the motor rotational speed (MRS), using the digital tachometer (TROY, TMR-F, Taipei, Taiwan); (C) shows the simulated blood flow (SBF) and displays the SBF measured by the flow meter (using the same sensor as (G)); (D) shows the simulated heart rate (SHR). A timer relay (ANLY, TRD-NC relay, Taipei, Taiwan) and a self-developed timer relay were used as the metering module to record the number of relay switches per minute; (E) is the check valve for flow resistance control of the simulated blood flow; (F) is the speed control motor and the timer relay to simulate the heart rate. The device models used for simulating the human heartbeat are the ES01 series 21K6RGN-AW2U speed control motor and ANLY’s TRD-NC timer relay (ANLY, TRD-NC relay, Taipei, Taiwan); (G) is the flow meter used for measuring the simulated blood flow circulation. The sensor used is a Nylon micro-meter (Vass Asia Sensormatic Corp, FM01, New Taipei, Taiwan) which is small in size and can detect a flow range of 1 to 60 L/Min ± 10%.

**Figure 7:**
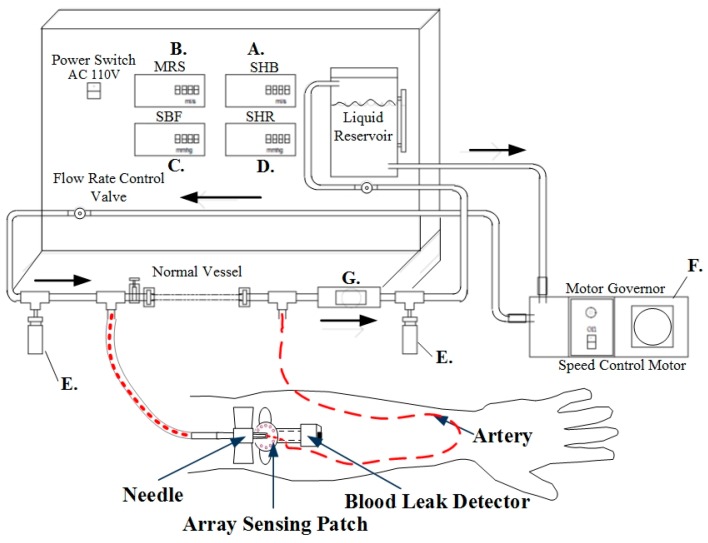
Design of blood circulation simulation system.

Figure 7 shows the system design. After setting the motor speed and timer relay, the adjustable flow valves were mounted before the blood outlet and before the blood inlet to the liquid reservoir. Blood flow is measured through the flow meter installed between the pipes and is shown in the system interface. To verify the system simulation results, before the simulated blood is returned to the liquid reservoir, Doppler ultrasound was utilized to measure the flow rate. The pseudo-blood fluid used in this system was mainly artificial blood or physiological saline. During experiments, corn starch can be added gradually to increase the fluid viscosity coefficient [15] and simulate different blood conditions.

**Figure 8:**
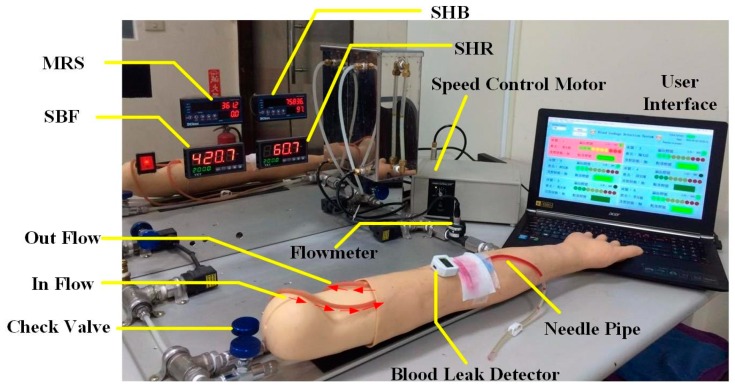
Blood circulation simulation system.

This simulation test cycle used a single-phase 110 V/60 Hz speed control motor (Orientalmotor, Speed Control Motor, New Taipei, Japan) with instantaneous stop, a variable speed range (60 Hz: 90–1600 r/min), and a timer relay (ANLY, TRD-NC relay, Taipei, Taiwan) to achieve the simulated heartbeat. The timer relay used in this study has an operating voltage of AC110/60 Hz. Its advantages are: a wide temperature range (−10 to +50 °C), a reset time of 0.2 s, and the switching time can be adjusted from 0.2 s–5 s, whichever is suitable for simulating human heartbeats within the range of 50 to 180 beats per minute. In order to verify the feasibility of the simulation system, it was combined with a flow meter, a Doppler ultrasound for flowrate measurement, and heart rate variability to verify the accuracy of the simulation system, as shown in Figure 8.

## 3. Experimental Design and Results

In order to verify the accuracy of the blood leakage detection and the stability of wireless transmission, a series of experimental tests were performed.

### 3.1. Single-Point Response Time

**Figure 9:**
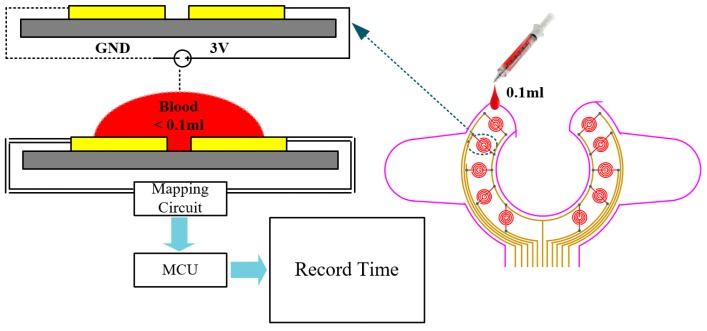
Schematic diagram of the multi-channel sensing experiment.

This experiment sequentially dropped simulated blood on individual sensing points to test the single-point detection sensitivity. First, the array sensor was placed flat on a level desk, and then the wearable blood leakage detector containing the Wi-Fi feature was connected and fixed on the connection terminal. After the start of the experiment, simulated blood was dropped sequentially on different sensing points, with experiments carried out 10 times on each sensing point, and the average response time for each sensing point was recorded, as shown in Figure 9. Experimental results showed that an average of 0.1 mL was needed for each sensing point. After testing each sensing point 10 times, the average and SD response time measured by the instruction cycles of the microprocessor was about 90 ± 5 ms.

### 3.2. Risk Level Experiment

In this study we made use of the “weight ratio” because each sensing point had its own contribution for the risk level. In order to verify whether the risk level function of our design is relative to that of an actual blood leakage, we performed two experiments to determine the validity of the risk level based on different weight ratios.

**Figure 10:**
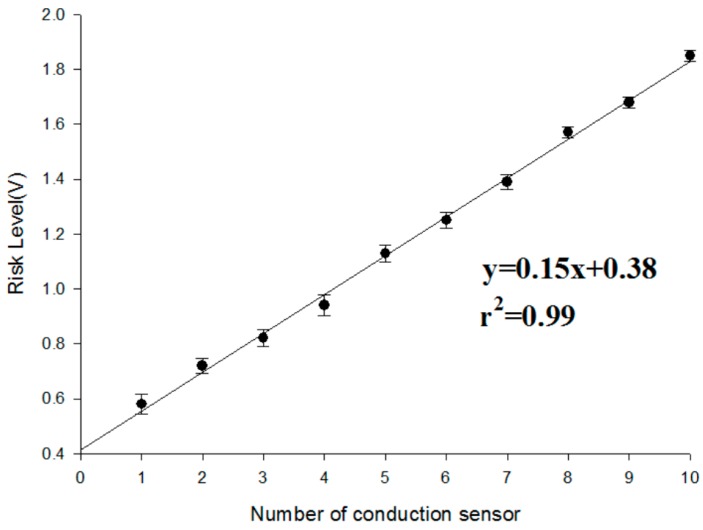
Experimental results for 10 individual conduction sensing points under the same weight ratio.

**Figure 11:**
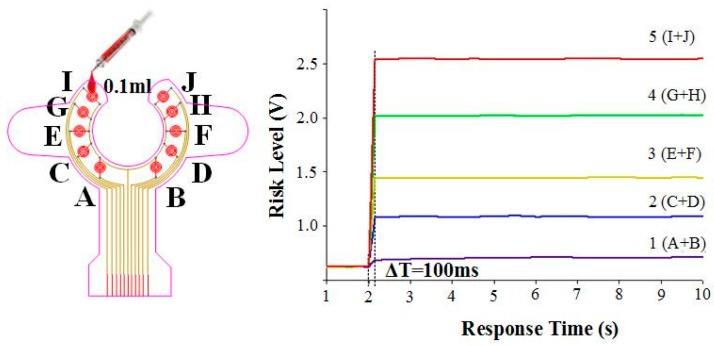
Experimental results for group sensing points with different weight ratios (mean value of risk level).

(a) First, in the experiment with the same weight ratio, we dropped 0.1 mL of blood on each sensing point in random order until all the sensor points were covered. This experiment was repeated 10 times. The results of the experiment are shown in Figure 10. With an increase in the number of conducting sensing points, the risk level voltage linearly increased. Based on the Student’s *t*-test analysis, they displayed significant differences between each other (*p* < 0.05) as shown in Table 1.
(b) Because each sensing point could have different weight ratios for the risk level, we designed a symmetrical weight ratio distribution using two parallel points to test different conditions of clinical blood leakage. Ten sensing points were divided into five groups, namely 1 (A + B), 2 (C + D), 3 (E + F), 4 (G + H), and 5 (I + J). The weight ratio was proportionally allocated in accordance with the distance from the tip of the needle. The weight ratio of 1 (A, B), which is the closest the needle, and 5 (I, H), which is the farthest from the needle, is 1:10. During the start of the experiment, the conduction for one group was done at fixed times to determine whether the difference in voltage level conforms to the weight ratio. Test results are shown in Figure 11. Group 1 (A and B), which is closer to the tip of the needle, shows a small change in voltage level difference after conduction. When the degree of blood leakage increased, Group 5 (I and J), which is located farthest from the tip of the needle, showed an increase in voltage level difference. The data ratio is about 1:10 and is consistent with the weight ratio values. The results indicated the risk level is highly reproducible and consistent with the blood leakage level. In addition, during conduction, the average response time of the system is less than 100 ms. Based on the Student’s t-test analysis, there were significant differences between the tests as shown in Table 2.

### 3.3. Circulatory System Simulation Test

**Figure 12:**
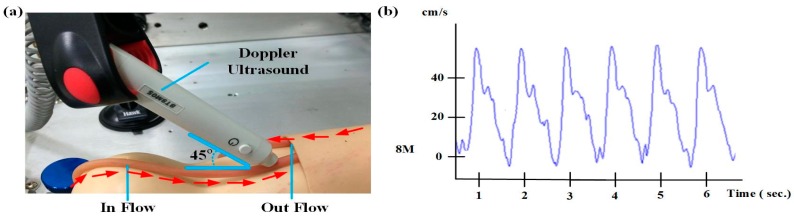
(**a**) The Doppler ultrasound placement angle for blood flow measurement and (**b**) simulated blood flow waveform.

Figure 12a shows the circulatory system simulation test used to verify the self-designed simulated human blood circulation system to attest to the correctness of the simulated human heart rate and blood flow. After the heart rate and flow rate adjustment, a hand-held Doppler ultrasound was used to measure the flow. Frequency was adjusted in accordance with different user frequencies such as 5 MHz (deep arterial and venous blood) or 8 and 10 MHz (superficial vessels). The Doppler ultrasound placement angle refers to [16] for velocity estimation. We chose a 45 degree angle to measure the blood flow in this study. The flow rate is 10 cm/s, based on the Smart-V-Link™ measurement results displayed in Figure 12b, and the flow rate is converted to the flow volume using Equation (2). The Doppler ultrasound made use of 8 MHz frequency to obtain a waveform that matches the setting of the simulated blood flow.

(2) $$Q=\frac{\pi D^{2}}{4}\times V$$

### 3.4. Gauze Diffusion Time Test for Different Flow Rates

**Figure 13:**
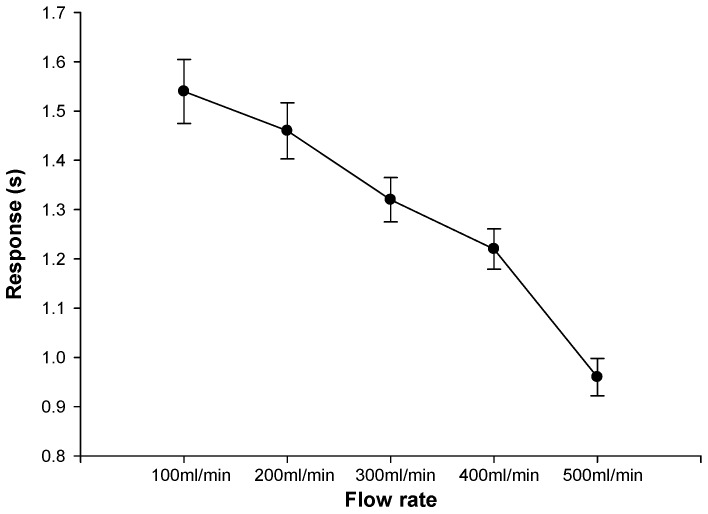
Test results of blood flow and blood leakage detection.

In our design, all of the sensor points were covered by gauze. This experiment was utilized to determine the required time to reach risk level 2 at different flow rates to check the effect of the blood spreading time in gauze. First, the needle was exposed to the simulated blood vessel, and then the array sensor was placed on top of the needle and covered with gauze to simulate an actual blood leakage situation. The experimental setting of the HD flow rate was set to 60 heartbeats per minute; the blood flow was set from 100 mL/min to 500 mL/min, with each flow being tested 10 times. When blood leakage reached the risk level, the system response time was recorded. Figure 13 shows the result of the test. Although the response time of each sensing point reaches 0.1 s, in actual HD treatment, a layer of gauze covers the array sensor. When blood leakage occurs, the gauze absorbs the blood and the array sensing time would reach an average of about 2 s. Subsequently, in the 10 experiments, the sensor was able to detect blood leakage and it correctly sent off a risk level warning. With a faster blood flow, the gauze absorption rate increases, and the overall system response time decreases.

### 3.5. Wireless Signal Attenuation Test of Wi-Fi

In order to verify the effective wireless transmission distance, an open outdoor space was chosen. Each Wi-Fi module was assigned a distance of 20, 40, and 60 m. Table 3 shows that data could be received at 60 m, and the received signal strength indicator (RSSI) is −83 dBm. If the RSSI is more than −80 dBm, the data loss rate of the proposed device is below 1%, thus meeting the requirement of this study. However, different coverings interfere with the actual experiments in hospitals. According to the actual dialysis ward size of about 10 × 30 m, the results of this experiment confirm the validity of a Wi-Fi wireless transmission system. Moreover, the results of this experiment are consistent with previous findings [17,18].

**Figure 14:**
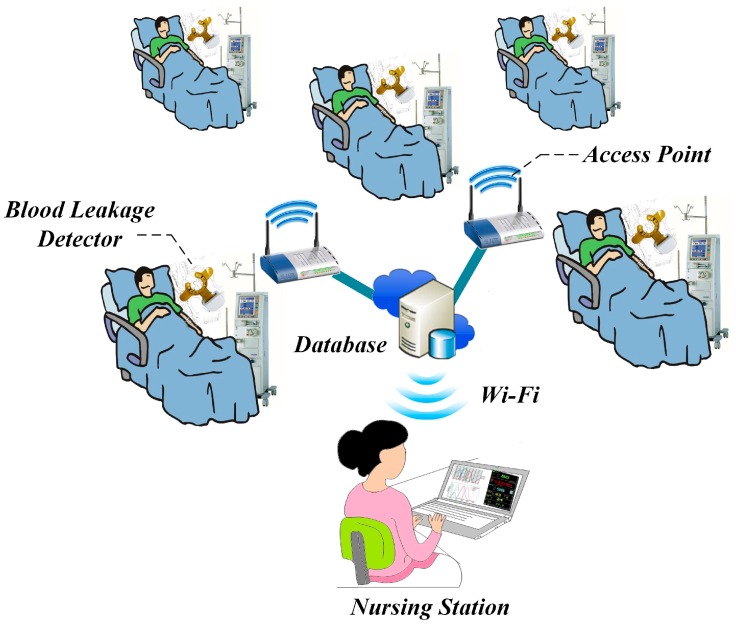
Schematic diagram of multi-bed Wi-Fi monitoring.

After the effective wireless transmission distance test, Figure 14 shows the experimental set-up of 10 devices connected to Wi-Fi. The transmission stability of the Wi-Fi system during multi-bed monitoring was tested. By setting up multiple APs, it could effectively monitor risk levels in each bed. Test results showed that the system could perform blood leakage level classification in multi-bed monitoring. At the same time, healthcare workers could view the status of all dialysis patients at a nursing station.

### 3.6. Battery Life Experiments

The device utilizes a compact rechargeable battery with design specifications of 3.7 V and 380 mAh, which will be beneficial for future miniaturization. In order to determine the usable time of the device and the wireless modules, a battery life experiment was executed. Experimental results showed that the CC3200 has higher power consumption in continuous Wi-Fi transmission, but without continuous transmission the power consumption is acceptable. Experiments indicated that the device can be operated for up to 9 h without Wi-Fi transmission. However, for continuous transmission at 10 Hz, the battery life can last up to 70 min, depending on the distance of the AP. The data loss rate is maintained within 1% error. Multiple APs of the Wi-Fi architecture enable each sensing device to transmit data first to the closest AP before relaying it to the main monitoring terminal. Overall, the proposed device could be used for around 6–8 h without frequent wireless transmissions with a clinical blood leakage scenario, which meets the requirement for clinical use.

## 4. Discussion and Conclusions

Previous studies and commercially available products use single-point blood leakage detection with Bluetooth wireless transmission. This study has successfully developed a wearable device using an array sensor patch combined with a mapping circuit and a Wi-Fi module. The multi-bed Wi-Fi architecture employed multiple-AP relay to reduce power consumption and increase the usage time of the device. In the future, IPv6 Internet Protocol could be combined to complete the two-way communication of a device while allowing better power consumption. The device is an independent system, and thus it could be used along with existing HD equipment. Although our design may be affected by sweat, based on the experience of clinicians, extensive sweating during dialysis is very rare because the environment of the dialysis ward is very comfortable and there is minimal patient activity. A symptom called “cold sweating” may appear in some patients who have autonomic neuropathy, diabetes or cardiovascular disorders during hemodialysis [19]. It is also an abnormal condition that needs to be monitored. Maybe our device could also have a contribution in this condition. This wearable VND blood leakage detection device is small in size and can be conveniently integrated to a multi-bed monitoring terminal and can be powered by a typical 3.7 V rechargeable battery.

The self-designed human circulatory simulation device, used for testing the blood diffusion time on the gauze under different blood flow rates, mimics the scenario in a real clinical therapy setting. Results showed that the proposed device response time was less than 2 s for a normal HD flow rate and the average response time was less than 1 s at a flow rate of 500 mL/min. This provides a fast monitoring system for HD therapy. Subsequently, the multi-bed Wi-Fi wireless transmission and battery life tests showed that in comparison to the actual dialysis processes which lasts about 6 to 8 h, the current device could continuously provide service for up to 8 h. Finally, results of the outdoor effective distance measurement and the multi-bed Wi-Fi wireless transmission showed that the current device has an effective distance of up to 60 m and a data loss rate of less than 1%. Although the Wi-Fi transmission model of the CC3200 consumes a large amount of energy [20], the usage time of the device was increased through modifications in different control modes to reduce the frequency of data link and transmission. WiFi is one of the commonly used wireless technologies in clinical studies which is also convenient to integrate into the hospital’s information system. Results from this project look forward to partnering with a low-power-consumption Wi-Fi module to increase the battery life cycle. It can be considered as a potential medical Internet of Things (IoT) device for clinical warning and evaluation to improve the medical service.

## Figures and Tables

**Table 1:** Statistical analyses results of the same sensing point weight ratio (*N* = 10).

Number of Conduction Sensor	Mean (V)	Number of Conduction Sensor	Mean (V)
1	0.58 ± 0.054	6	1.25 ± 0.051
2	0.72 ± 0.052	7	1.39 ± 0.045
3	0.82 ± 0.048	8	1.57 ± 0.042
4	0.94 ± 0.051	9	1.68 ± 0.021
5	1.13 ± 0.057	10	1.85 ± 0.025

**Table 2:** Statistical analysis results of risk level in the different weight ratios.

Group	Mean ± SD
1 (A + B)	0.18 ± 0.041
2 (C + D)	0.52 ± 0.039
3 (E + F)	0.94 ± 0.031
4 (G + H)	1.48 ± 0.045
5 (I + J)	1.92 ± 0.052

All groups have significant differences with each other.

**Table 3:** Result of the frequency propagation test.

	Distance (m)	Wi-Fi (dBm)	Data Loss (%)
Detector	20 m	−72	0%
	40 m	−76	0%
	60 m	−83	<1%

Transmission power is 0 dBm (1 mW).

## Abbreviations

The following abbreviations are used in this manuscript:

HD	Hemodialysis
CKD	Chronic kidney disease
ESRD	End-stage renal disease
VND	Venous needle dislodgement
FPCB	Flexible Printed Circuit Board
SHB	Simulation heart beat
MRS	Motor rotational speed
SBF	Simulation blood flow
SHR	Simulation heart rate
IoT	Internet of Things
